# Dynamics of Forward and Backward Translocation of mRNA in the Ribosome

**DOI:** 10.1371/journal.pone.0070789

**Published:** 2013-08-09

**Authors:** Ping Xie

**Affiliations:** Key Laboratory of Soft Matter Physics and Beijing National Laboratory for Condensed Matter Physics, Institute of Physics, Chinese Academy of Sciences, Beijing, China; University of Lethbridge, Canada

## Abstract

Translocation of the mRNA-tRNA complex in the ribosome, which is catalyzed by elongation factor EF-G, is one of critical steps in the elongation cycle of protein synthesis. Besides this conventional forward translocation, the backward translocation can also occur, which can be catalyzed by elongation factor LepA. However, the molecular mechanism of the translocation remains elusive. To understand the mechanism, here we study theoretically the dynamics of the forward translocation under various nucleotide states of EF-G and the backward translocation in the absence of and in the presence of LepA. We present a consistent explanation of spontaneous forward translocations in the absence of EF-G, the EF-G-catalyzed forward translocations in the presence of a non-hydrolysable GTP analogue and in the presence of GTP, and the spontaneous and LepA-catalyzed backward translocation. The theoretical results provide quantitative explanations of a lot of different, independent experimental data, and also provide testable predictions.

## Introduction

During the elongation cycle of protein synthesis, mRNA and tRNA are moved through the ribosome by the dynamic process of translocation, which takes place via two steps [1], [2]. First, peptidyl-tRNA and deacylated tRNA are transited between classical (A/A and P/P sites, respectively) and hybrid (A/P and P/E sites, respectively) states. Then, catalyzed by elongation factor EF-G and GTP, the two tRNAs that are coupled with mRNA via codon-anticodon interaction are transited from the hybrid to post-translocation (P/P and E/E sites) state. However, it was observed that the translocation can occur spontaneously, albeit quite slowly and inefficiently, in the absence of EF-G and GTP [3]–[7]. Addition of EF-G and GDPNP (a nonhydrolyzable analog of GTP) to the solution containing the pre-translocation ribsosomal complex promotes significantly the translocation to the post-translocation state [8]–[13]. When GDPNP is replaced with GTP, the translocation rate is increased further [8]–[13]. Moreover, it was shown that EF-G hydrolyzes GTP before the translocation of mRNA and tRNA [8]–[12].

Besides the conventional forward translocation from pre- to post-translocation state, it was intriguingly found that, in some contexts, spontaneous and efficient conversion from the post- to pre-translocation state can also occur in the absence of translational factors [14], [15]. It was demonstrated that EF4 (or LepA) – another translational factor present in bacteria, mitochondria and chloroplasts – can catalyze this backward translocation [16]–[19].

However, the molecular mechanism of these translocations remains elusive. For example, how do the spontaneous forward and backward translocations take place? How does EF-G in combination with GTP or GDPNP catalyze forward translocation? Why does EF-G.GTP have a greater potency in catalyzing forward translocation over EF-G.GDPNP? How does LepA catalyze backward translocation? Here, to address these questions, we theoretically study the dynamics of forward translocation under various nucleotide states of EF-G (in the absence of EF-G, with the binding of EF-G.GDPNP and with the binding of EF-G.GTP), as well as the dynamics of backward translocation in the absence and presence of LepA. We give a consistent and quantitative explanation of a lot of different, independent experimental data. The studies have important implications for understanding the mRNA translocation mechanism.

## Methods

We study the dynamics of forward and backward translocation based mainly on the following pieces of experimental evidence and argument.

Evidence (i) – The peptidyl transfer, i.e., deacylated tRNA bound to the 30S P site and/or peptidyl-tRNA bound to the 30S A site, results in the ribosome being in a “labile” state, allowing the relative rotation between two ribosomal subunits, with the two conformations called non-ratchet and ratchet (or hybrid) states [10], [20]–[24].

Evidence (ii) – The binding of EF-G.GTP shifts the equilibrium toward the hybrid state of the labile ribosome [10], [20]–[24].

Evidence (iii) – The 50S E site has a high affinity for deacylated tRNA and the 50S P site has a specific interaction with the peptidyl moiety [25], [26].

Argument (iv) – In the presence of a tRNA anticodon stem-loop bound to the 30S A site, the binding of EF-G.GTP reduces the interaction of the 30S subunit with the mRNA-tRNA complex, and after GTP hydrolysis the unlocking of the ribosome further reduces the interaction of the 30S subunit with the mRNA-tRNA complex. In other words, with a tRNA anticodon stem-loop bound to the 30S A site, the affinity of the 30S subunit for the mRNA-tRNA complex is dependent on the nucleotide state of EF-G: high affinity without EF-G, low affinity after ribosomal unlocking (in EF-G.GDP.Pi state), and intermediate affinity with EF-G.GTP. Without a tRNA anticodon stem-loop bound to the 30S A site, the interaction of the 30S subunit with the mRNA-tRNA complex is independent of the nucleotide state of EF-G. The argument is inferred from the following available experimental evidence. A tRNA anticodon stem-loop bound to the 30S A site is minimally required for translocation of mRNA [27]. EF-G activates the translocation in the presence of GDPNP, whereas the translocation rarely occurs in the absence of EF-G [13], [28]. Moreover, the binding of EF-G.GDPNP promotes mRNA back-slippage [28], implying the reduction of the interaction of the 30S subunit with the mRNA-tRNA complex. After EF-G.GTP hydrolysis, smaller conformational changes in EF-G cause a shift of domain IV toward the decoding center, which could detach the mRNA-tRNA complex from the decoding center [29], [30]. It is noted here that the effect of EF-G on the interaction of the 30S subunit with the mRNA-tRNA complex is via the interaction of EF-G with the tRNA bound to the 30S A site.

### Equations for Transitions between Non-ratchet and Ratchet States

**Figure 1:**
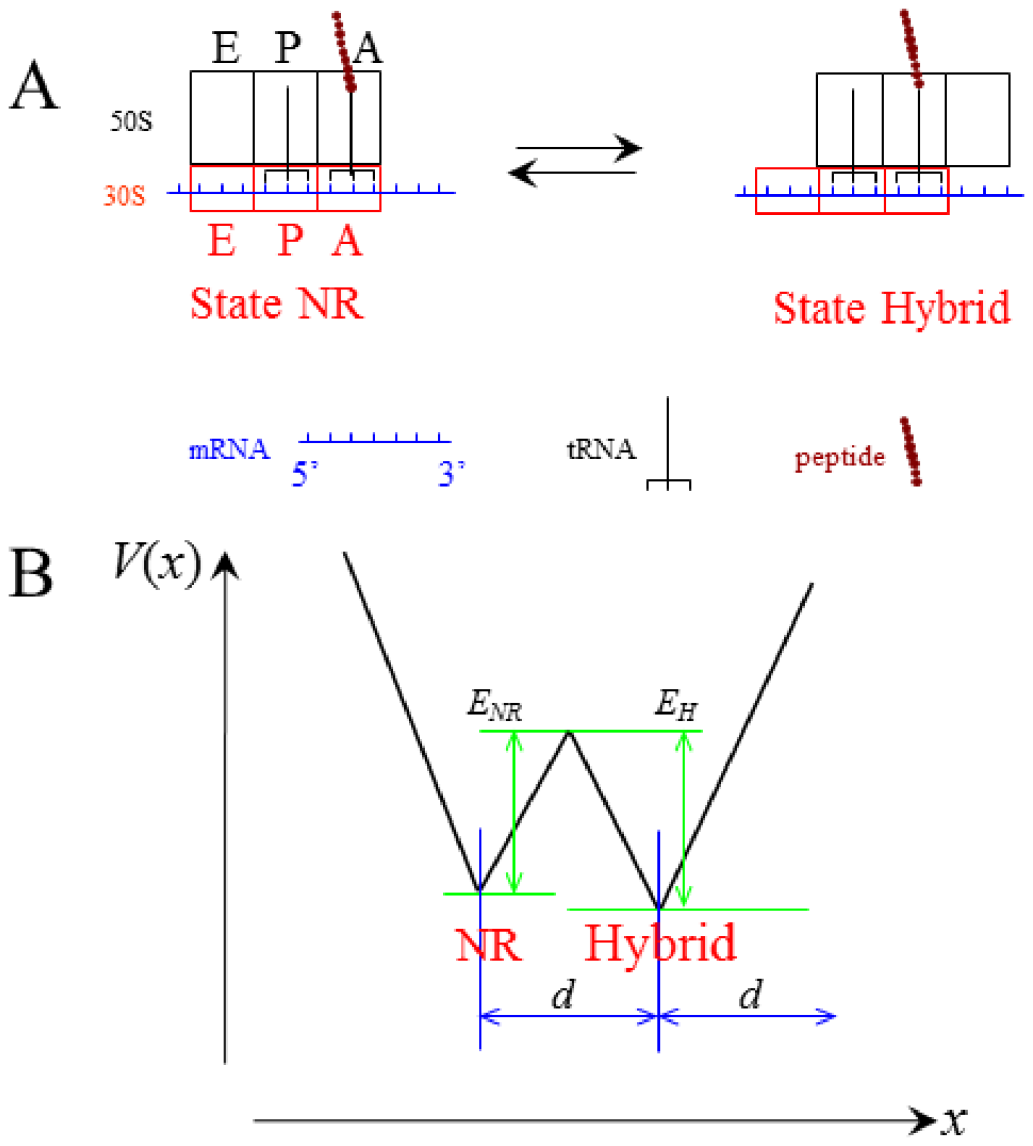
The labile state of ribosome with deacylated tRNA bound to the 30S P site and peptidyl-tRNA bound to the 30S A site. (a) Schematic of transition from the classical non-ratchet state (State NR) to hybrid state (State hybrid) and vice versa. (b) Potential *V*(*x*) that characterizes the transition between the classical non-ratchet and hybrid states.

Consider the deacylated tRNA bound to the 30S P site and the peptidyl-tRNA bound to the 30S A site, as shown in Fig. 1a. Thus, according to evidence (i), the ribosome is now in the labile state and can transit from the classical non-ratchet (left, Fig. 1a) to hybrid (right, Fig. 1a) state and vice versa. Denoting by *E_NR_* the energy barrier for transition from the classical non-ratchet to hybrid state and *E_H_* the energy barrier for transition from the hybrid to classical non-ratchet state, potential *V*(*x*) that characterizes the motion of the 30S subunit relative to the 50S subunit is approximately shown in Fig. 1b and the Langevin equation to describe the motion is described as follows
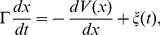
(1)where 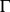 is the frictional drag coefficient on the motion of the 30S subunit relative to the 50S subunit and 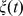 represents the fluctuating Langevin force, with 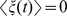 and 
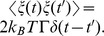
 The choice of the value of 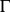 in our calculation is discussed as follows. For simplicity, we consider the ribosomal 30S subunit as a sphere of radius *r* = 5 nm and take the viscosity of the aqueous cytoplasm 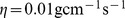 (see Discussion). From the Stokes-Einstein law, we have 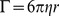 = 9.4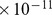 kg 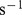. From Eq. (1), the mean first-passage time for transition from the classical non-ratchet (left, Fig. 1a) to hybrid (right, Fig. 1a) state can be calculated by [31]

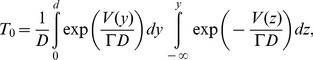
(2)where 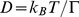 and d = 2 nm is the moved distance of the 30S subunit relative to the 50 S subunit [1]. With potential V(x) given in Fig. 1b, we finally derive
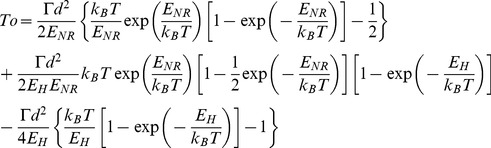
(3)


It is noted that when *E_NR_* and *E_H_*>>*k_B_T*, Eq. (3) becomes 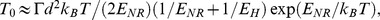 i.e., *T*_0_ approximately has a linear relation with 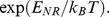


The mean time for transition from the hybrid (right, Fig. 1a) to classical non-ratchet (left, Fig. 1a) state can also be calculated by Eq. (3) but with *E_NR_* and *E_H_* being replaced by *E_H_* and *E_NR_*, respectively.

### Equations for Forward Translocation

**Figure 2:**
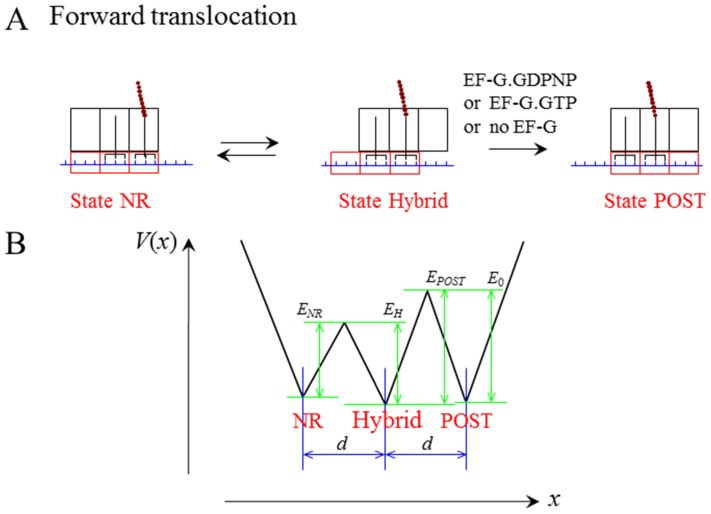
Forward translocation. (a) Schematic of transition from pre-translocation state, including the classical non-ratchet state (State NR) and hybrid state (State hybrid), to post-translocation state (State POST). (b) Potential *V*(*x*) that characterizes the transition from the pre- to post-translocation state.

In Fig. 1, due to the high affinity of the 30S subunit for the mRNA-tRNA complex, it is implicitly assumed that the mRNA-tRNA complex is fixed to the 30S subunit during transition from the hybrid to non-ratchet state. As we will show below, this is a good approximation for the case in the absence of EF-G. Considering that the mRNA-tRNA complex can also be moved relative to the 30S subunit, the ribosomal complex can transit from the hybrid state either to the classical non-ratchet state or to the post-translocation state, as shown in Fig. 2a. Now the potential *V*(*x*) that characterizes the state transitions is approximately shown in Fig. 2b, where *E_POST_* represents the energy barrier for transition from the hybrid (middle, Fig. 2a) to post-translocation (right, Fig. 2a) state and *E*_0_ represents the energy barrier for the reverse transition. The Langevin equation to describe the state transitions can still be described by Eq. (1).

To be consistent with the procedure used in the experiments to measure the spontaneous mRNA translocation time in the absence of EF-G [7], the mean mRNA translocation time is defined as the mean time for the ribosomal complex to transit from the classical non-ratchet (left, Fig. 2a) to hybrid (middle, Fig. 2a) to post-translocation (right, Fig. 2a) state. Thus, the mean mRNA translocation time can be calculated by [31]

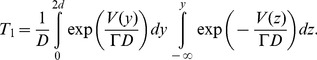
(4)


With potential *V*(*x*) given in Fig. 2b, from Eq. (4) we finally obtain 
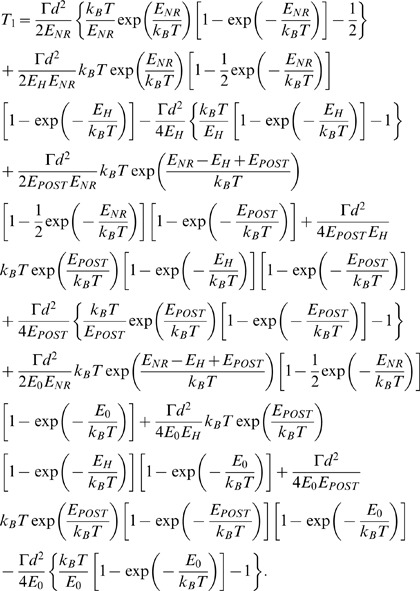
(5)


It is noted that when *E_NR_*, *E_H_*, *E_POST_* and *E*_0_>> *k_B_T*, the expansion of Eq. (5) does not give an obviously useful form.

After addition of EF-G and GDPNP or GTP to the solution containing pre-translocation complex, the complex is most of time in the hybrid state. Thus, the mean mRNA translocation time can be approximately calculated by [31]

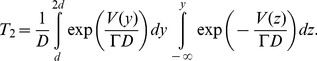
(6)


With potential *V*(*x*) given in Fig. 2b, from Eq. (6) we finally obtain
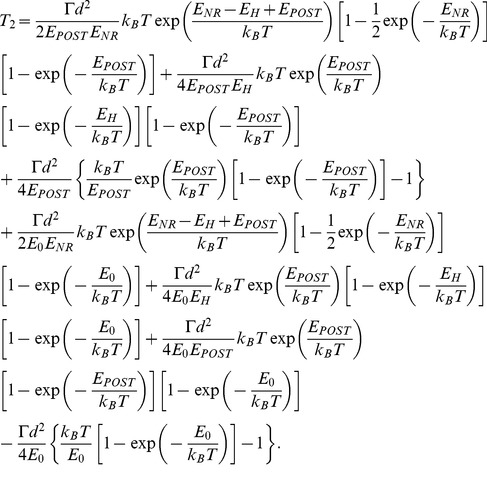
(7)


It is noted that when *E_NR_*, *E_H_*, *E_POST_* and *E*_0_>> *k_B_T*, Eq. (7) becomes
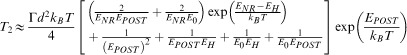
(8)


From Eq. (8), it is seen that *T*_2_ approximately has a linear relation with 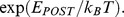


## Results

### Determination of Energy Barriers for Transitions between Non-ratchet and Ratchet States of the Labile Ribosome

In this section, we determine energy barriers *E_NR_* and *E_H_* in the labile state of the ribosome with the deacylated tRNA bound to the 30S P site and the peptidyl-tRNA bound to the 30S A site (Fig. 1). We use smFRET data of Cornish et al. [24] to determine values of *E_NR_* and *E_H_*. The smFRET data showed that for the pre-translocation ribosome with peptidyl-tRNA analog N-Ac-Phe-tRNA^Phe^ bound to the 30S A site and deacylated tRNA^fMet^ bound to the 30S P site, the rate of transition from the classical non-ratchet to hybrid state is *k* ^(*F*)^ = 0.27 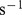 and the rate of reverse transition is *k* ^(*B*)^ = 0.19 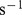 [24]. Using Eq. (3) we obtain that when *E_NR_* = 23.87*k_B_T* and *E_H_* = 24.24*k_B_T*, the transition times 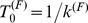
and 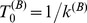 are in agreement with the experimental data [24]. This implies that in the absence of EF-G, the labile state of the ribosome with the peptidyl-tRNA bound to the 30S A site and the deacylated tRNA bound to the 30S P site approximately has *E_NR_* = 23.87*k_B_T* and *E_H_* = 24.24*k_B_T*. It is noted here that in order to ensure that the two tRNAs, driven by the thermal noise, cannot move from A/A and P/P sites to P/P and E/E sites in the classical non-ratchet state (left, Fig. 2a), it is required that the affinity of the 30S subunit in pre-translocation non-ratchet state for the mRNA-tRNA complex should be larger than *E_NR_* = 23.87*k_B_T*.

Available experimental data indicated that the binding of EF-G.GDPNP shifts the equilibrium toward the hybrid state [evidence (ii)]. Here we also use smFRET data of Cornish et al. [24] to determine the energy change resulting from this equilibrium biasing. The smFRET data showed that when EF-G.GDPNP is bound to the pre-translocation complex with deacylated tRNA^fMet^ bound to the 30S P site, the rate of transition from classical non-ratchet to hybrid state is increased by about 2.33-fold, implying that *E_NR_* is reduced by about 0.85*k_B_T*, while the rate of the reverse transition is decreased by about 10-fold, implying that *E_H_* is increased by about 2.30*k_B_T*. In other words, the binding of EF-G.GDPNP induces the decrease of energy barrier *E_NR_* by about 0.85*k_B_T* and the increase of energy barrier *E_H_* by about 2.30*k_B_T*, implying that the binding of EF-G.GDPNP shifts the equilibrium toward the ratchet conformation by an energy decrease of about 3.15*k_B_T*. Thus, after the binding of EF-G.GDPNP the energy barriers for the ribosomal complex as shown in Fig. 1a, *E_NR_* and *E_H_* are changed to *E_NR_* = 23.02*k_B_T* and *E_H_* = 26.54*k_B_T*.

In the following studies of mRNA translocation time we will take *E_NR_* = 23.87*k_B_T* and *E_H_* = 24.24*k_B_T* in the absence of EF-G and the effect of the binding of EF-G.GDPNP on energy barriers *E_NR_* and *E_H_* as shown above. Since different buffer conditions or contexts would have different values of *E_NR_* and *E_H_*, it is interesting to study the effect of variations of *E_NR_* and *E_H_* on the mRNA translocation time, as presented in Text S1 and Figures S1– S5, where it is shown that the variations of *E_NR_* and *E_H_* only have small effects on the mRNA translocation time.

### Forward Translocation in the Absence of EF-G

As determined above, *E_NR_* = 23.87*k_B_T* and *E_H_* = 24.24*k_B_T* in the absence of EF-G (Table 1). Considering the specific affinity, 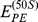, of the 50S E site for deacylated tRNA and the 50S P site for the peptidyl moiety [evidence (iii)], the energy barrier *E_H_* can be written as 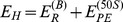, where 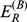 represents the intrinsic energy barrier for the ribosome to rotate from the ratchet to non-ratchet conformation if the affinity 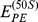 is not included. By fitting to the single molecule experimental data [32], it has been determined that the specific affinity of the 50S E site for deacylated tRNA and the 50S P site for peptidyl-tRNA is about 9*k_B_T* [33]. Taking 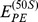 = 9*k_B_T*, we have 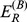 = 15.24*k_B_T*. Based on argument (iv), the energy barrier *E_POST_* is calculated by
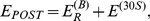
(9)where 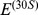 now represents the affinity of the 30S subunit in hybrid state for the mRNA-tRNA complex in the absence of EF-G. It is noted here that since both the transition from State Hybrid to State NR and the transition from State Hybrid to State POST (Fig. 2) are induced by the reverse ribosomal rotation from the rotated to non-rotated conformation, the intrinsic energy barrier of reverse ribosomal rotation (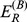) is the same in both transitions.

**Table 1:** Summary of energy barriers during forward translocation.

Parameters	no EF-G	EF-G.GDPNP	EF-G.GTP		
			Case I	Case II	Case III
*E_NR_*	23.87*k_B_T*	23.02*k_B_T*	23.87*k_B_T*	23.02*k_B_T*	24.72*k_B_T*
*E_H_*	24.24*k_B_T*	26.54*k_B_T*	24.24*k_B_T*	26.54*k_B_T*	21.94*k_B_T*
*E_POST_*	33.91*k_B_T*	23.33*k_B_T*	15.24*k_B_T*	17.54*k_B_T*	12.94*k_B_T*
*E*_0_	29.09*k_B_T*	29.09*k_B_T*	29.09*k_B_T*	29.09*k_B_T*	29.09*k_B_T*
*E* ^(30S)^	18.67*k_B_T*	5.79*k_B_T*	0	0	0

**Figure 3:**
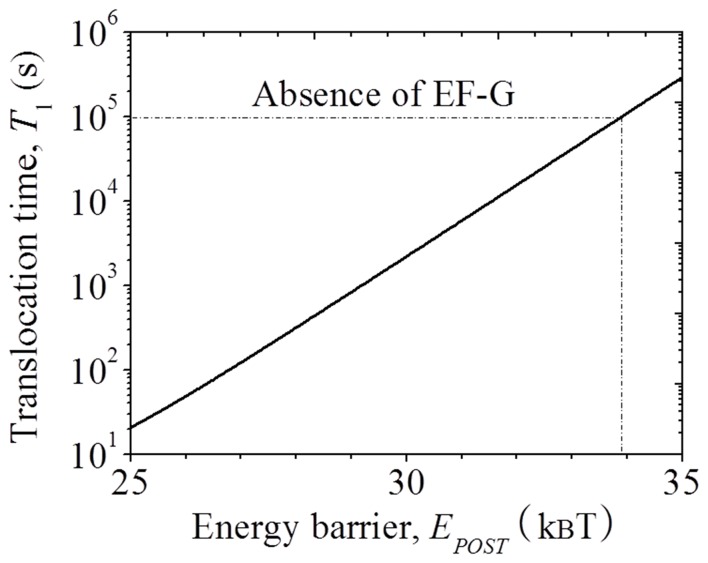
Results of forward mRNA translocation time *T*_1_ as a function of energy barrier *E_POST_*, which are calculated by using Eq. (5), with *E_NR_* = 23.87 *k_B_T* and *E_H_* = 24.24 *k_B_T* (corresponding to the case in the absence of EF-G).

As we will show below, when the energy barrier *E*_0_ is about 29.09*k_B_T*, the spontaneous backward translocation rate is consistent with the available experimental data [14]. Thus, in the following calculations of forward mRNA translocation time, we take *E*_0_ = 29.09*k_B_T* (Table 1). In fact, as it is noted from Eqs. (5) and (7), the forward mRNA translocation time is insensitive to the value of *E*_0_ (see also Text S2 and Figure S6). Thus, taking other values of *E*_0_ has only a small effect on the mean mRNA translocation time. With *E_NR_* = 23.87*k_B_T* and *E_H_* = 24.24*k_B_T* (Table 1), using Eq. (5) we calculate mRNA translocation time *T*_1_ as a function of energy barrier *E_POST_*, with the results shown in Fig. 3. The available experimental data showed that the spontaneous mRNA translocation rate *k*_1_ = 4–6×10^-4^min^-1^ [7], [14], giving *T*_1_=1/*k*_1_≈ 1×10^5^ s. From Fig. 3, it is seen that this value of *T*_1_ = 1 ×10^5^ s corresponds to *E_POST_* = 33.91*k_B_T* (Table 1). It is noted here that although *E_POST_* is smaller than *E*_0_, the conversion of the hybrid sate to post-translocation state can still occur, but with the maximal fraction of the post-translocation state converted being much small than unity, as indicated by the experimental data [7]. As just obtained above, we have 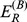 = 15.24*k_B_T*. Thus, from Eq. (9) we obtain 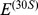 = 18.67*k_B_T* (Table 1), which is smaller than that (>23.87*k_B_T*) in the non-ratchet state (see above section), consistent with the proposal by McGarry et al. [34] that movement of deacylated tRNA from the classical P/P state to hybrid P/E state destabilizes codon–anticodon interaction.

Since before transition to post-translocation state (State POST), the ribosomal complex would take many cycles of transition from hybrid state (State Hybrid) to classical non-ratchet state (State NR) and vice versa, it is interesting to calculate the cycling number here. Using Eq. (3) it is calculated that the transition time from State Hybrid to State NR is about 5.26 s while the backward transition from State NR to State Hybrid is about 3.70 s, giving one cycling time of about 8.96 s. If the transition from State Hybrid to State NR is not allowed, using Eq. (3) it is calculated that the transition from State Hybrid to State POST is about 45931 s. Thus, it is easily obtained that for the case without EF-G, it takes about 6034 cycles of transition from State Hybrid to State NR and vice versa before transition to State POST.

### Forward Translocation with the Binding of EF-G.GDPNP

**Figure 4:**
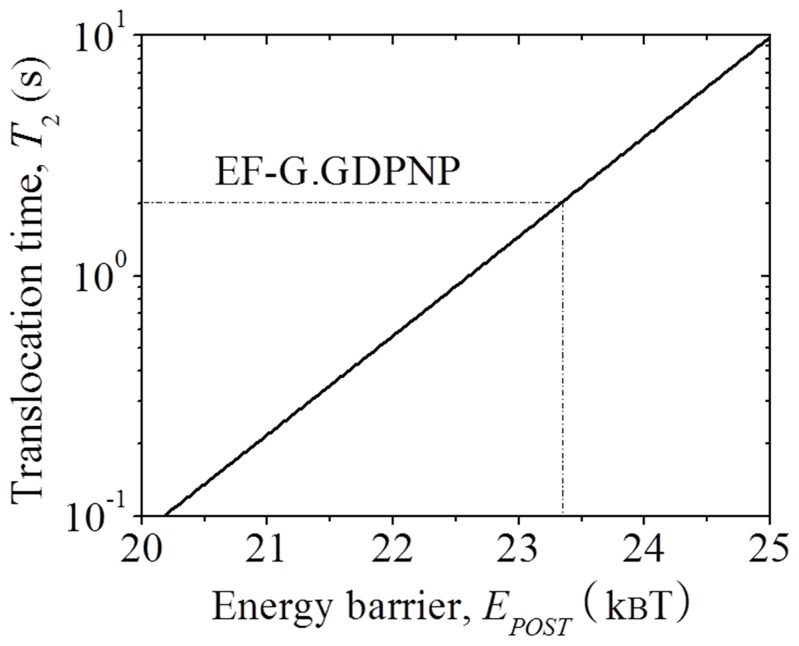
Results of forward mRNA translocation time *T*_2_ as a function of energy barrier *E_POST_*, which are calculated by using Eq. (7), with *E_NR_* = 23.02 *k_B_T* and *E_H_* = 26.54 *k_B_T* (corresponding to the case with binding of EF-G.GDPNP).

As shown above, after the binding of EF-G.GDPNP the energy barriers *E_NR_* and *E_H_* are changed to *E_NR_* = 23.02*k_B_T* and *E_H_* = 26.54*k_B_T* (Table 1). With these values of *E_NR_* and *E_H_*, using Eq. (7) we calculate mRNA translocation time *T*_2_ as a function of energy barrier *E_POST_*, with the results shown in Fig. 4. The available experimental data showed that the mRNA translocation rate *k*_2_ = 0.5 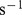 [8], giving 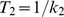 = 2 s. From Fig. 4, it is seen that this value of *T*_2_ = 2 s corresponds to *E_POST_* = 23.33*k_B_T* (Table 1).

Based on evidence (ii) and argument (iv), after the binding of EF-G.GDPNP the energy barrier *E_POST_* is calculated by
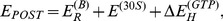
(10)where 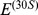 now represents the affinity of the 30S subunit in hybrid state for the mRNA-tRNA complex with the binding of EF-G.GDPNP and 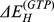 represents the increase of energy barrier *E_H_* induced by the binding of EF-G.GDPNP. As determined above, 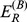 = 15.24*k_B_T* and 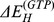 = 2.30*k_B_T*. Thus, from Eq. (10) we obtain 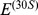 = 5.79*k_B_T* (Table 1), implying that the binding of EF-G.GDPNP or EF-G.GTP induces the affinity of the 30S subunit for the mRNA-tRNA complex to decrease from about 18.67*k_B_T* to about 5.79*k_B_T* (Table 1) or decrease by about 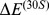 = 12.88*k_B_T*.

### Forward Translocation with the Binding of EF-G.GTP

Hydrolysis of EF-G.GTP to EF-G.GDP.Pi induces ribosomal unlocking, detaching mRNA-tRNA complex from the decoding center [argument (iv)]. Thus, the affinity of the 30S subunit for the mRNA-tRNA complex becomes *E* ^(30S)^ 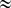 0 (Table 1). To study the translocation with *E* ^(30S)^ 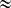 0, we consider three cases for the effect of the ribosomal unlocking on shifting the equilibrium between non-ratchet and ratchet conformations.

**Figure 5:**
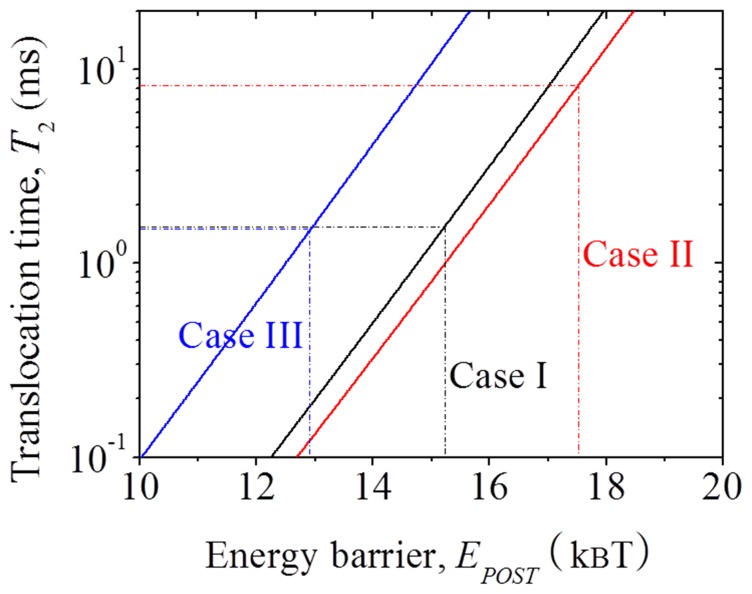
Results of forward mRNA translocation time *T*_2_ as a function of energy barrier *E_POST_* after the binding of EF-G.GTP, which are calculated by using Eq. (7), with *E_NR_* = 23.87*k_B_T* and *E_H_* = 24.24 *k_B_T* (black line, Case I), *E_NR_* = 23.02 *k_B_T* and *E_H_* = 26.54*k_B_T* (red line, Case II), and with *E_NR_* = 24.72*k_B_T* and *E_H_* = 21.94*k_B_T* (blue line, Case III).

In Case I, the ribosomal unlocking has no effect on the equilibrium between non-ratchet and ratchet conformations, as in the absence of EF-G. Thus, we have *E_POST_* = 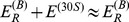 = 15.24*k_B_T* (Table 1). With *E_NR_* = 23.87*k_B_T* and *E_H_* = 24.24*k_B_T* (Table 1), using Eq. (7) we calculate mRNA translocation time *T*_2_ as a function of energy barrier *E_POST_*, with the results shown in Fig. 5 (Case I). From Fig. 5 (Case I), it is seen that *T*_2_ = 1.57 ms at *E_POST_* = 15.24*k_B_T*. This value of *T*_2_ = 1.57 ms is much shorter than the time of GTP hydrolysis followed by ribosomal unlocking, 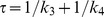 = 32.57 ms (see Figure S7), where *k*_3_ = 250 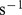 and *k*_4_ = 35 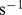 are taken from available biochemical data [35], . Thus, the mRNA translocation time is mainly determined by time 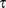. It is noted here that the mRNA translocation rate 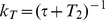 = 29.29 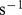 is consistent with the available experimental value (of about 25 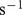) by Rodnina et al. [8].

In Case II, the ribosomal unlocking shifts the equilibrium toward the ratchet conformation, as EF-G.GTP state does. Thus, we have *E_POST_* = 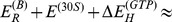
17.54*k_B_T* (Table 1). With *E_NR_* = 23.02*k_B_T* and *E_H_* = 26.54*k_B_T* (Table 1), using Eq. (7) we calculate mRNA translocation time *T*_2_ as a function of energy barrier *E_POST_*, with the results shown in Fig. 5 (Case II). From Fig. 5 (Case II), it is seen that *T*_2_ = 8.63 ms at *E_POST_* = 17.54*k_B_T*. This value of *T*_2_ = 8.43 ms is also much shorter than the time of GTP hydrolysis followed by ribosomal unlocking, 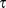 = 32.57 ms. Thus, even if the ribosomal unlocking has the effect of shifting the equilibrium toward the ratchet conformation, as EF-G.GTP state does, the mRNA translocation time is also mainly determined by time 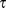. Note here that the mRNA translocation rate 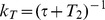 = 24.39 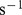 is also consistent with the available experimental value of about 25 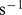 [8].

In Case III, the ribosomal unlocking shifts the equilibrium toward the non-ratchet conformation, which is contrary to Case II but with the same magnitudes of the effect on the energy barriers *E_NR_*, *E_H_* and *E_POST_*. Thus, the energy barriers *E_NR_* and *E_H_* are now changed to *E_NR_* = 24.72*k_B_T* and *E_H_* = 21.94*k_B_T* (Table 1), and *E_POST_* = 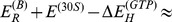
12.94*k_B_T* (Table 1). With *E_NR_* = 24.72*k_B_T* and *E_H_* = 21.94*k_B_T* (Table 1), using Eq. (7) we calculate mRNA translocation time *T*_2_ as a function of energy barrier *E_POST_*, with the results shown in Fig. 5 (Case III). It is seen that *T*_2_ = 1.52 ms at *E_POST_* = 12.94*k_B_T*. Interestingly, it is noted that this value of *T*_2_ = 1.52 ms is very close to that (1.57 ms) for Case I, which can be understood as follows. On the one hand, the reduction of the energy barrier *E_POST_* due to the equilibrium shifting toward the non-ratchet conformation facilitates the transition from the hybrid to post-translocation state, decreasing the mean mRNA translocation time. On the other hand, the shifting of equilibrium toward the non-ratchet conformation facilitates the transition from the hybrid to classical non-ratchet pre-translocation state, inducing the increase of the mean time for transition from the hybrid to post-translocation state. The two opposite effects on the mRNA translocation thus tend to cancel one another. In other words, the shifting of equilibrium toward the non-ratchet conformation has nearly no effect on mRNA translocation after the ribosomal unlocking.

Taken together, our data indicate that whether the ribosomal unlocking has no effect or has the effect of shifting the equilibrium between non-ratchet and ratchet conformations, after ribosomal unlocking the small 30S subunit would rapidly ratchet backward with respect to the large 50S subunit, which is consistent with the biochemical [35] and structural [29] data. This reverse ribosomal rotation induces the mRNA translocation by one codon, which is consistent with the experimental data of Ermolenko and Noller [13]. Moreover, the translocation time is mainly determined by the time of GTP hydrolysis followed by ribosomal unlocking.

### Backward Translocation in the Absence of Translational Factors

**Figure 6:**
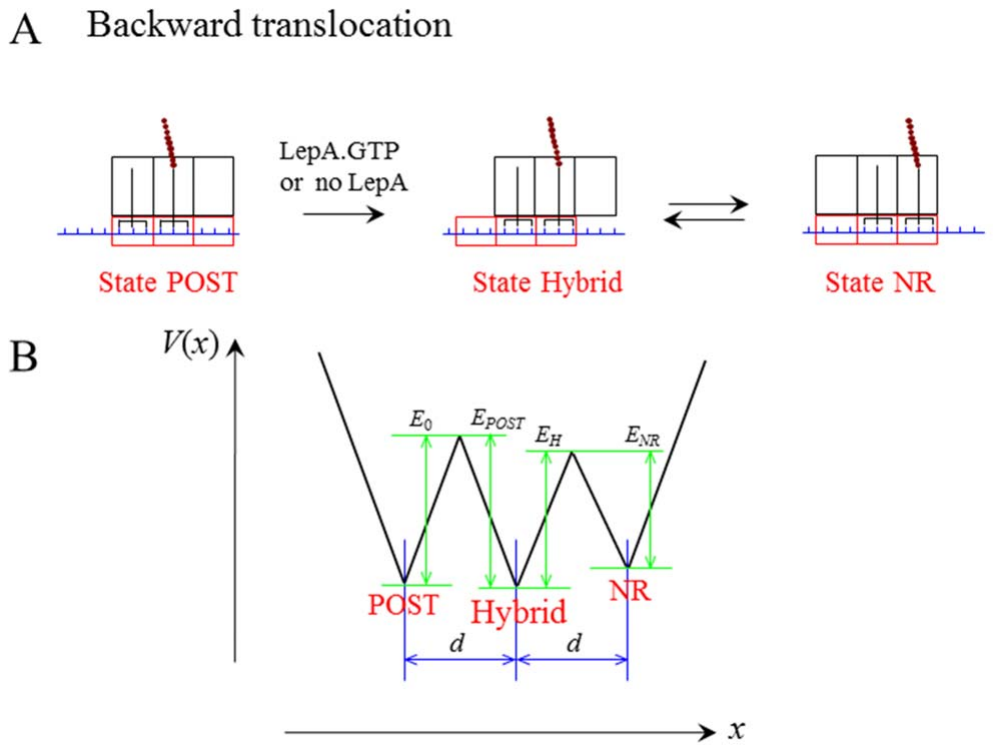
Backward translocation. (a) Schematic of transition from post- (State POST) to pre-translocation state, including the classical non-ratchet state (State NR) and hybrid state (State hybrid). (b) Potential *V*(*x*) that characterizes the transition from the pre- to post-translocation state.

Consider the post-translocation ribosomal complex with the deacylated tRNA bound to the E site and the peptidyl-tRNA bound to the P site, as shown in Fig. 6a (left). Thus, according to evidence (i), the ribosome is now in the non-labile state. However, it is noted that the ribosome in the non-labile state does not mean that the ribosome is in the fixed conformation. Rather, the ribosome can still transit between the non-ratchet and ratchet conformations but with much lower transition rates than in the labile state.

Before the study of the spontaneous backward translocation, we first focus on the transition between the non-ratchet and ratchet conformations of the non-labile vacant ribosome (i.e., one lacking tRNA). The smFRET data of Cornish et al. [24] showed that for the vacant ribosome, the rate of transition from non-ratchet to ratchet conformation is *k* ^(*F*)^ = 0.015 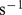 and the rate of reverse transition is *k* ^(*B*)^ = 0.02 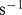. Using Eq. (3) we obtain that when *E_NR_* = 26.99*k_B_T* and *E_H_* = 26.69*k_B_T*, the transition times 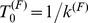 and 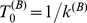 are in agreement with the experimental data [24]. This implies that the non-labile vacant ribosome approximately has *E_NR_* = 26.99*k_B_T* and *E_H_* = 26.69*k_B_T*. As determined above, the labile ribosome has *E_NR_* = 23.87*k_B_T*. Thus, the free energy to fix the conformation in the non-labile non-ratchet state is about 3.12*k_B_T* larger than that in the labile state.

**Figure 7:**
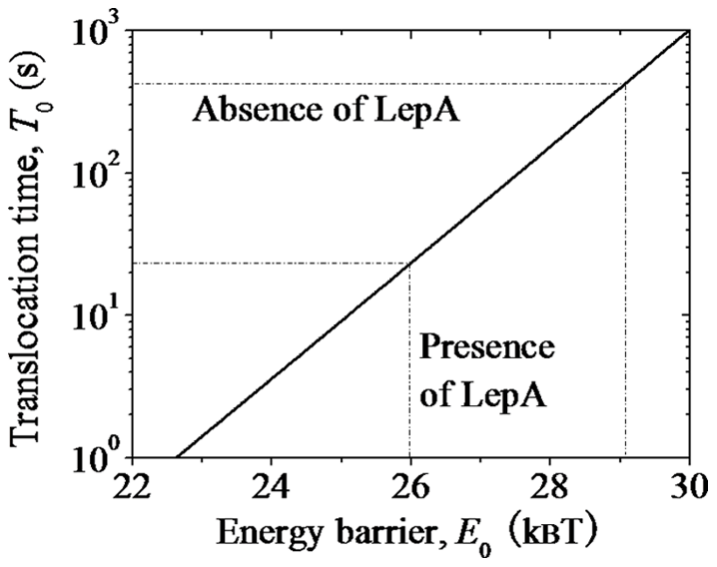
Results of backward mRNA translocation time *T*_0_ as a function of energy barrier *E*_0_, which are calculated by Eq. (3) but with *E_NR_* and *E_H_* being replaced by *E*_0_ and *E_POST_*, respectively. *E_POST_* = 33.91*k_B_T*.

Now, we study the spontaneous backward translocation time of the ribosome bound by two tRNAs, as shown in Fig. 6a (left). The backward mRNA translocation time can be calculated by Eq. (3) but with *E_NR_* and *E_H_* being replaced by *E*_0_ and *E_POST_* (Fig. 6b), respectively. After the backward translocation, the hybrid state (middle, Fig. 6a) becomes that as shown in the middle of Fig. 2a, with *E_POST_* = 33.91*k_B_T* (see above). The calculated results of the spontaneous backward translocation time *T*_0_ versus *E*_0_ are shown in Fig. 7. The available experimental data showed that the spontaneous backward translocation rate is *k = *0.14 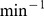 [14], giving 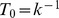 = 428.57 s. From Fig. 7 it is seen that this value of *T*_0_ = 428.57 s corresponds to *E*_0_ = 29.09*k_B_T* (Table 2). This implies that at least in some contexts of Shoji et al. [14], the energy barrier for transition from the post- to pre-translocation state is about 29.09*k_B_T*.

**Table 2:** Summary of energy barriers in posttranslocation state.

Parameters	no elongation factor	LepA
*E*_0_	29.09*k_B_T*	25.97*k_B_T*
*E* ^(30S)^	2.10*k_B_T*	2.10*k_B_T*

After transition to the hybrid state (middle, Fig. 6a), since the deacylated tRNA is now bound to the 30S P site the ribosome becomes labile. Then the ribosomal complex transits easily between the hybrid (middle, Fig. 6a) and classical non-ratchet (right, Fig. 6a) states, as studied above. Since *E_POST_* = 33.91*k_B_T* is larger than *E*_0_ = 29.09*k_B_T*, the pre-translocation state is thermodynamically favored over the post-translocation state, consistent with the experimental data [14].

It is noted that the energy barrier *E*_0_ = 29.09*k_B_T* of the post-translocation state is 2.10*k_B_T* larger than *E_NR_* = 26.99*k_B_T* of the vacant ribosome. This indicates that the affinity of the 30S subunit for the mRNA-tRNA complex in the post-translocation state is about 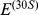 = 2.10*k_B_T* (Table 2), which is smaller than the affinity of about 5.79*k_B_T* in the pre-translocation state bound by EF-G.GTP (see above).

**Figure 8:**
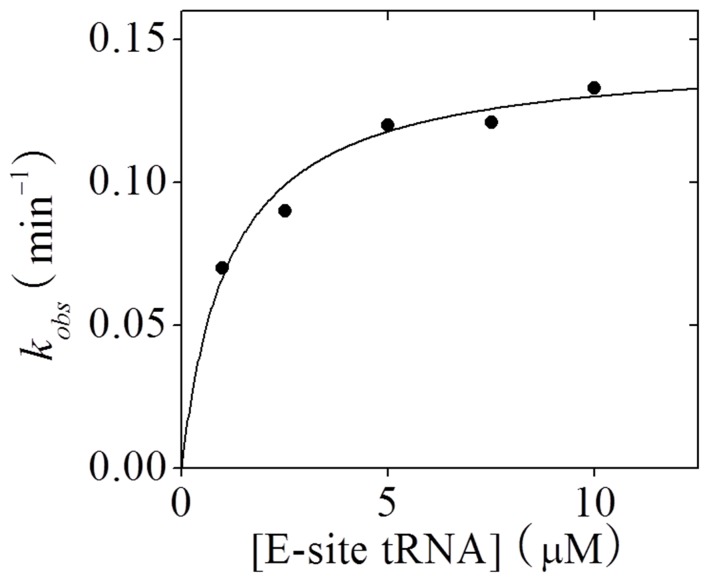
Spontaneous backward mRNA translocation rate (*k_obs_*) versus concentration of E-site tRNA. Line represents the theoretical results and filled circles are experimental data taken from Shoji et al. [14].

Experimental data showed that when only peptidyl-tRNA is bound to the 30S P site, the spontaneous backward translocation cannot occur or cannot be detected experimentally [14], [15]. Here, based on our calculations we give explanations of this phenomenon (see Text S3 and Figure S8). Moreover, the experimental data of spontaneous backward translocation rate versus concentration of E-site tRNA can be quantitatively explained, which is shown as follows. Since for the post-translocation ribosome with only peptidyl-tRNA bound to the 30S P site, only after deacylated tRNA binds to the E site can the backward translocation occur or be detected, the observed backward translocation time can be calculated by 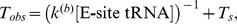 where 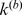 is the binding rate of deacylated tRNA to the E site, [E-site tRNA] the concentration of E-site tRNA and *T_s_* the backward translocation time of the ribosome with the deacylated tRNA bound to the E site and the peptidyl-tRNA bound to the P site, as studied above. Then, the observed backward translocation rate 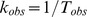 has the form
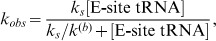
(11)where 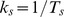. Note that the dependence of *k_obs_* on concentration of E-site tRNA has the Michaelis-Menten form. Using Eq. (11) the experimental data of *k_obs_* versus [E-site tRNA] [14] can be fitted well, as shown in Fig. 8, with fitted parameters 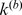 = 0.125 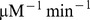 and *k_s_ = *0.145 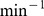. This value of *k_s_* is consistent with the backward translocation rate when two tRNAs are present [14].

### Backward Translocation in the Presence of LepA and GTP

It has been shown that translational factor LepA has the ability to catalyze the backward translocation by binding to the post-translocation ribosomal complex [16]–[19]. However, how LepA catalyzes the backward transition from the post- to pre-translocation state is unclear. Here, we only consider that the binding of LepA to the post-translocation state has the effect of inducing the non-labile ribosome to be labile. Under this effect, we study the dynamics of backward translocation.

As determined above, in the labile state, the energy barrier for transition from the non-ratchet to hybrid state is *E_NR_* = 23.87*k_B_T*. Then, in the labile state, the energy barrier for transition from the post-translocation to hybrid state is 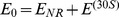 = 25.97*k_B_T* (Table 2), where 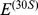 = 2.10*k_B_T* as determined in above section. From Fig. 7 it is seen that at *E*_0_ = 25.97*k_B_T*, the backward translocation time *T*_0_ = 22.75 s, giving the translocation rate *k* = 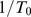 = 2.64 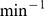, which is about 20-fold of the spontaneous backward translocation rate (about 0.14 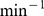). This rate of 2.64 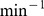 is consistent with that deduced from the experimental data [16], implying that only the effect of LepA.GTP on altering the non-labile state of the ribosome is sufficient to give an efficient conversion of the ribosomal complex from the post- to pre-translocation state.

After transition to the hybrid state, the ribosomal complex can transit between the hybrid and classical non-ratchet state. After the release of the hydrolysis products Pi and LepA.GDP, EF-G.GTP binds to the pre-translocation complex, thus the translation elongation proceeding.

### Predicted Results for Forward Translocation with the Binding of EF-G.GDPNP

**Figure 9:**
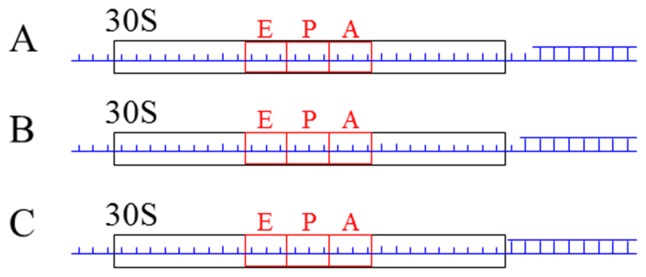
Schematic of the 30S subunit complexed mRNA containing one (a), two (b) and three (c) base pairs in the codon which is immediately adjacent to the mRNA entry channel in the 30S subunit.

When the deacylated tRNA and peptidyl-tRNA are bound to the 30S P site and A site, respectively, of the ribosome complexed with the single-stranded mRNA, the forward mRNA translocation time with the binding of EF-G.GDPNP has been studied before (Fig. 4), giving a quantitative explanation of the available experimental data [8]. In order to further test our theoretical studies by future experiments, we present some predicted results that are related to ribosome translation through the duplex region of mRNA [37]. We consider that the two tRNAs are bound to the 30S P and A sites of the ribosome complexed with mRNA containing a region of duplex, as shown in Fig. 9 where, in the codon, which is immediately adjacent to the mRNA entry channel in the 30S subunit and is downstream away from the A-site codon by three codons [32], there is one (Fig. 9a), two (Fig. 9b) and three mRNA bases (Fig. 9c) forming base pairs with bases of another mRNA strand. We study the forward translocation time of the mRNA with the binding of EF-G.GDPNP.

In Fig. 9, the transition from the hybrid to post-translocation state requires unwinding of one (Fig. 9a), two (Fig. 9b) and three (Fig. 9c) mRNA base pairs [32], [37]. Using the nearest-neighbor thermodynamic model for RNA duplex stability [38], it is estimated that the base-pairing free energy of an RNA base pair is *E_bp_* = 3*k_B_T*. Thus, we have *E_POST_* = 26.33*k_B_T* (Fig. 9a), 29.33*k_B_T* (Fig. 9b) and 32.33*k_B_T* (Fig. 9c) in potential *V*(*x*) shown in Fig. 2a. The other energy barriers in potential *V*(*x*) are the same as those for the case of single-stranded mRNA. With *E_NR_* = 23.02*k_B_T* and *E_H_* = 26.54*k_B_T* (see Table 1), using Eq. (7) we obtain that at *E_POST_* = 26.33*k_B_T*, 29.33*k_B_T* and 32.33*k_B_T*, the translocation time *T*_2_ = 35.24 s, 636.54 s and 11675.54 s, giving the translocation rate of about 0.028 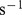, 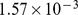 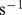 and 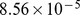 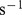, respectively. These imply that the mRNA translocation rates in Fig. 9a, b and c are about 0.056-fold, 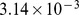-fold and 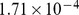-fold of the translocation rate of about 0.5 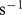 measured by Rodnina et al. [8] when the ribosome is complexed with the single-stranded mRNA.

## Discussion

We show that in the absence of EF-G or in the presence of EF-G.GDPNP, the mRNA translocation time is determined by reverse ribosomal rotation time from the hybrid to post-translocation state (Fig. 2a), which is in turn mainly determined by the affinity (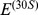) of the 30S subunit in hybrid state for the mRNA-tRNA complex. For a high affinity 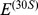, the ribosome would make many cycles of transition from the hybrid to classical non-ratchet state and vice versa before transition to the post-translocation state. However, in the presence of EF-G.GTP, since the reverse ribosomal rotation time from the hybrid to post-translocation state is much shorter than the time of GTP hydrolysis followed by ribosomal unlocking, the mRNA translocation time is mainly determined by the latter time. It is interesting to note here that the argument that the affinity 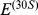 is a critical factor dictating the EF-G-independent mRNA translocation rate can also give an explanation of the experimental data showing that the omission of two 30S interface proteins S12 and S13 yields ribosomal particles that undergo efficient translocation in the absence of EF-G [39], implying that the two proteins S12 and S13 play major role in the interaction of the 30S subunit with the mRNA-tRNA complex.

Interestingly, we show that whether the ribosomal unlocking has no effect or has the effect of shifting the equilibrium between non-ratchet and ratchet conformations, the mRNA translocation time is mainly determined by the time of GTP hydrolysis followed by ribosomal unlocking. More interestingly, we show that the shifting of the equilibrium toward the non-ratchet conformation has nearly no effect on the mRNA translocation after the ribosomal unlocking. Thus, we prefer the following dynamic character. After peptidyl transfer, the labile ribosome can transit thermodynamically between the classical non-ratchet and hybrid states. The binding of EF-G.GTP shifts the thermodynamic equilibrium toward the hybrid state. Then, after hydrolysis of EF-G.GTP to EF-G.GDP.Pi the ribosomal unlocking either induces the ribosome to return to the thermodynamic equilibrium between the two ribosomal conformations, as before EF-G.GTP binding, or still shifts the thermodynamic equilibrium toward the ratchet conformation, as after EF-G.GTP binding.

After peptidyl transfer and before EF-G.GTP binding, the affinity of the 30S subunit in classical non-ratchet state for the mRNA-tRNA complex is larger than 23.87*k_B_T*. In the hybrid state, the affinity of the 30S subunit in for the mRNA-tRNA complex is reduced to about 18.67*k_B_T* (Table 1). The binding of EF-G.GTP induces the affinity to decrease from about 18.67*k_B_T* to about 5.79*k_B_T* (i.e., the affinity is decreased by about 12.88*k_B_T*) (Table 1), and after GTP hydrolysis the ribosomal unlocking induces the affinity to decrease further by about 5.79*k_B_T*, i.e., with nearly no affinity (Table 1). After translocation to the post-translocation state, the affinity of the 30S subunit for the mRNA-tRNA complex is changed to be about 2.10*k_B_T* (Table 2), which is smaller than the affinity of about 5.79*k_B_T* in the pre-translocation hybrid state bound by EF-G.GTP. Since the affinity of the ribosome for deacylated tRNA is composed of the affinity of the 50S E site and that of the 30S site, the larger value of 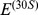 in the hybrid state than in the post-translocation state would result in the dissociation rate of deacylated tRNA in the hybrid state to be much smaller than in the post-translocation state, which is consistent with previous theoretical [40] and experimental [41] data.

We show that the free energy to fix non-ratchet conformation of the non-labile ribosome is about 3.12*k_B_T* larger than that of the labile ribosome. The occurrence of the spontaneous backward translocation in the absence of translational factor LepA is via overcoming the free energy to fix non-ratchet conformation of the non-labile ribosome plus the affinity of the 30S subunit for the mRNA-tRNA complex. We further show that if the binding of LepA to the post-translocation state has the effect of inducing the non-labile ribosome to be labile, the obtained LepA-catalyzed backward translocation rate is consistent with the experimental data [16], implying that only this effect of LepA is sufficient to give an efficient conversion of the ribosomal complex from the post- to pre-translocation state.

It should be mentioned that in our calculations, we have simply assumed the ribosomal 30S subunit as a sphere of radius *r* = 5 nm and taken the viscosity of the aqueous cytoplasm 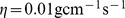, giving a frictional drag coefficient 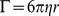 = 9.4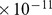 kg 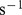. Considering that the real shape of the 30S subunit deviates from a sphere, the correct value of 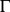 could be different from the above value [42]. Since the transition times (*T*_0_, *T*_1_, *T*_2_) are all proportional to 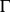 [see Eqs. (3), (5) and (7)], anything that affects our estimate of 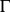 (the shape and dimension of the ribosomal subunit and the viscosity 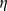) therefore corresponds to a uniform time dilation. As some experiments showed that the viscosity of the aqueous cytoplasm does not differ from that of water [43], [44], in the calculation we have taken the viscosity of the aqueous cytoplasm to be the same as that of water, i.e., 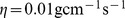. If we take value of 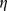 to be four-fold of that in pure water, as measured in other experiments [45], we would obtain a four-fold increase of the transition time, giving the energy barriers (*E_NR_*, *E_H_*, *E_POST_* and *E*_0_) to be about 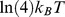 = 1.38*k_B_T* smaller than those given in the present work.

In our calculations of forward translocation catalyzed by EF-G.GDPNP, we used a translocation rate of *k*_2_ = 0.5 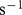 from Rodnina et al. [8] to obtain the energy barrier *E_POST_* = 23.33*k_B_T*. Some other experiments determined the EF-G.GDPNP-catalyzed translocation rate to be *k*_2_ = 1 to 6 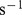 [11]–[13], giving 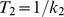 = 0.17 to 1 s. From Fig. 4, it is seen that values of *T*_2_ in this range correspond to *E_POST_* = 20.75*k_B_T* to 22.61*k_B_T*, which is 0.28*k_B_T* to 2.58*k_B_T* smaller than 23.33*k_B_T* determined with *k*_2_ = 0.5 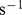. With *E_POST_* = 20.75*k_B_T* to 22.61*k_B_T*, from Eq. (9) we obtain 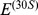 = 3.21*k_B_T* to 5.07*k_B_T*, implying that the binding of EF-G.GTP induces the affinity of the 30S subunit for the mRNA-tRNA complex to decrease from about 18.67*k_B_T* to about 3.21*k_B_T* to 5.07*k_B_T*.

In order to further test our analyses by future experiments, we provide predicted results on the forward mRNA translocation time with the binding of EF-G.GDPNP in the ribosome bound by the mRNA containing one, two and three base pairs in the codon which is downstream away from the A-site codon by three codons. We show that the translocation rates of the mRNA containing one, two and three base pairs are respectively about 0.056-fold, 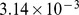-fold and 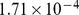-fold of the rate (about 0.5 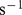) for the case of single-stranded mRNA. These results can be easily tested by future experiments.

## Supporting Information

Figure S1
**Spontaneous mRNA translocation time** ***T*** **_1_ as a function of 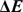 in the absence of EF-G.**
(TIF)

Figure S2
**mRNA translocation time** ***T*** **_2_ as a function of 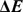 after the binding of EF-G.GDPNP.**
(TIF)

Figure S3
**mRNA translocation time** ***T*** **_2_ as a function of 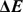 after the binding of EF-G.GTP for Case I that the ribosomal unlocking has no effect on the equilibrium between non-ratchet and ratchet conformations.**
(TIF)

Figure S4
**mRNA translocation time** ***T*** **_2_ as a function of 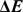 after the binding of EF-G.GTP for Case II that the ribosomal unlocking shifts the equilibrium toward the ratcheted conformation, as EF-G.GTP state does.**
(TIF)

Figure S5
**mRNA translocation time** ***T*** **_2_ as a function of 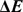 after the binding of EF-G.GTP for Case III that the ribosomal unlocking shifts the equilibrium toward the non-ratcheted conformation, which is contrary to Case II.**
(TIF)

Figure S6 **mRNA translocation time** ***T*** **_1_ as a function of** ***E*** **_0_.** ***E_NR_*** **= 23.02** ***k_B_T*** **,** ***E_H_*** **= 26.54** ***k_B_T*** **and** ***E_POST_*** **= 23.33** ***k_B_T*** **.**
(TIF)

Figure S7 **Kinetic scheme of EF-G.GTP-catalyzed mRNA translocation.**
(TIF)

Figure S8 **Schematic illustrations of two possible cases for backward translocation when only peptidyl-tRNA is bound to the P site.**
(TIF)

Text S1 **Effect of variations of** ***E_NR_*** **and** ***E_H_*** **on forward mRNA translocation time.**
(DOC)

Text S2 **Effect of variation of** ***E*** **_0_ on forward mRNA translocation time.**
(DOC)

Text S3 **Backward translocation cannot occur or cannot be detected when only peptidyl-tRNA is bound to the P site.**
(DOC)
