# Supplementary figures and images for: Dynamics of Forward and Backward Translocation of mRNA in the Ribosome
Source: PLoS One. 2013 Aug 9;8(8):e70789. doi: 10.1371/journal.pone.0070789 (PMC3739767; doi:10.1371/journal.pone.0070789)

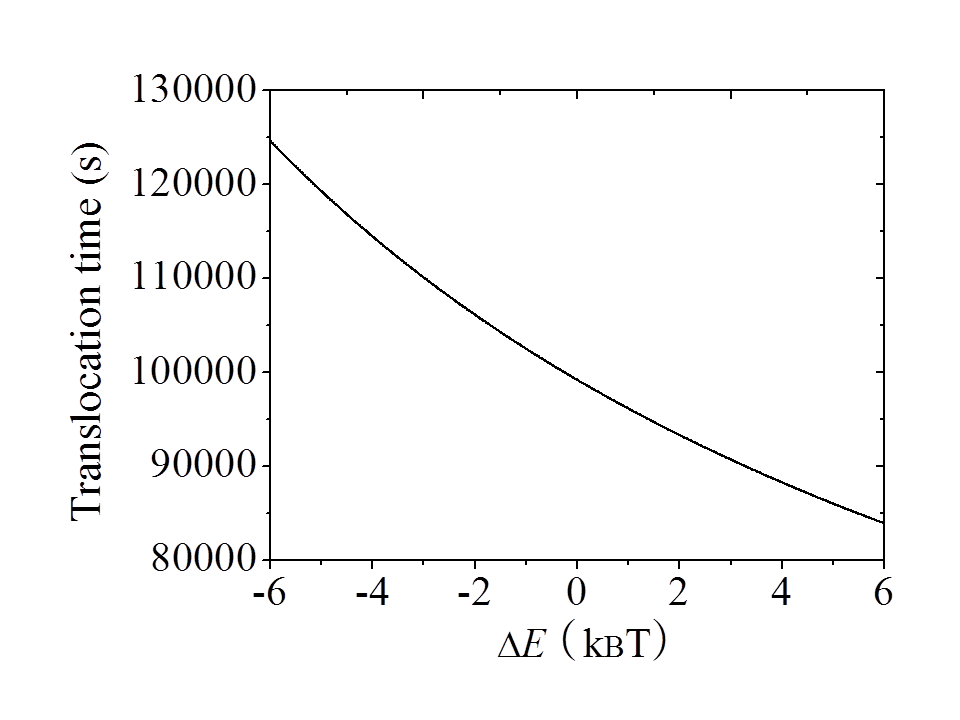

Supplement: Figure S1 — Spontaneous mRNA translocation time T 1 as a function of in the absence of EF-G. (TIF) [file pone.0070789.s001.tif]

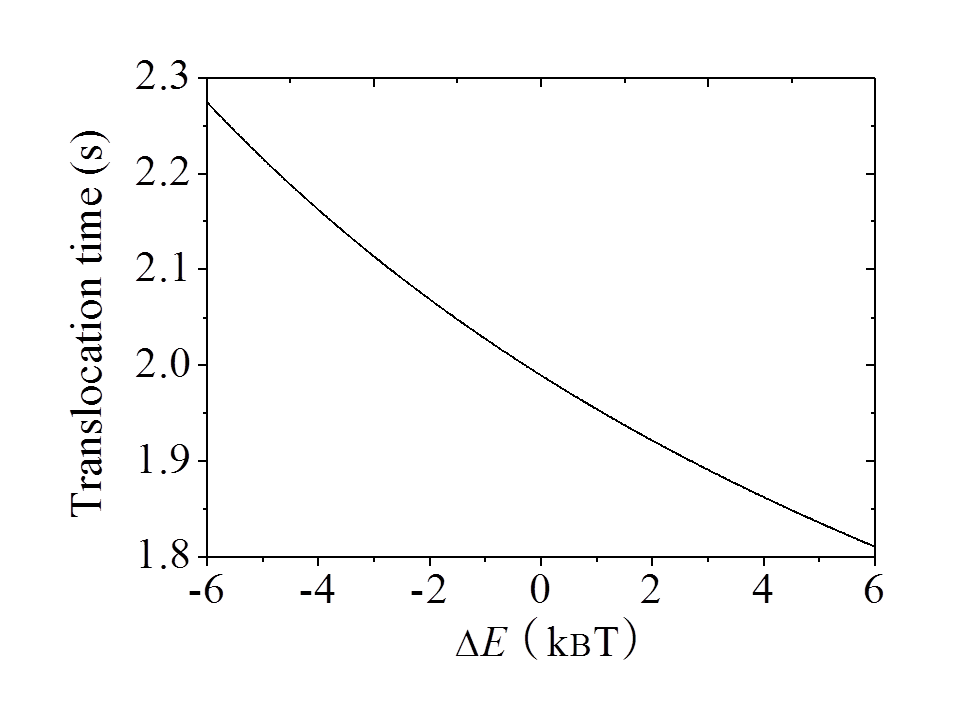

Supplement: Figure S2 — mRNA translocation time T 2 as a function of after the binding of EF-G.GDPNP. (TIF) [file pone.0070789.s002.tif]

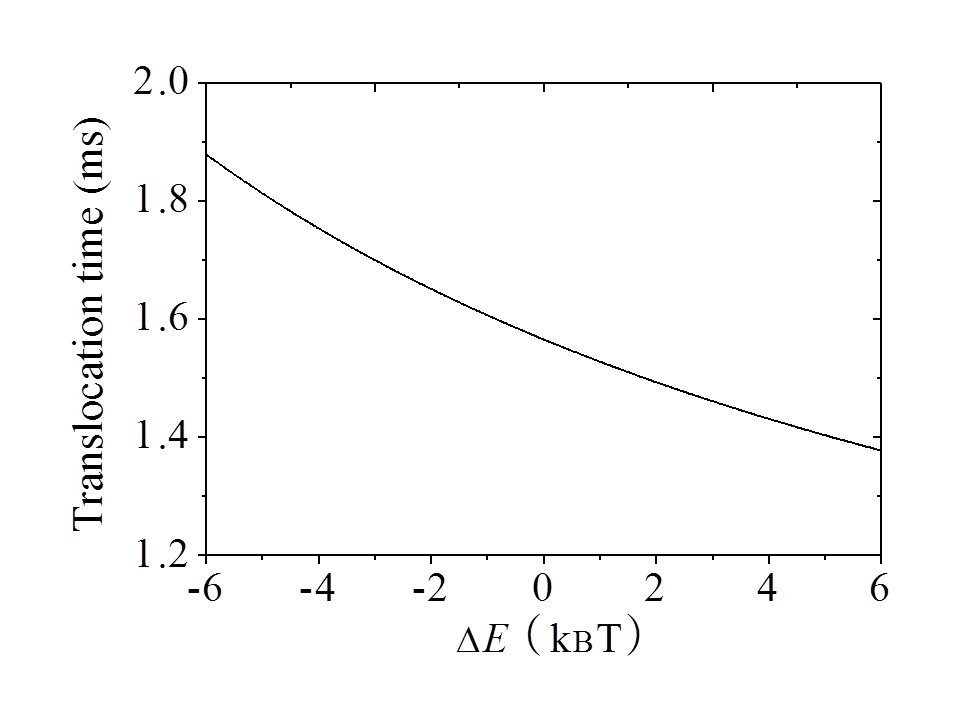

Supplement: Figure S3 — mRNA translocation time T 2 as a function of after the binding of EF-G.GTP for Case I that the ribosomal unlocking has no effect on the equilibrium between non-ratchet and ratchet conformations. (TIF) [file pone.0070789.s003.tif]

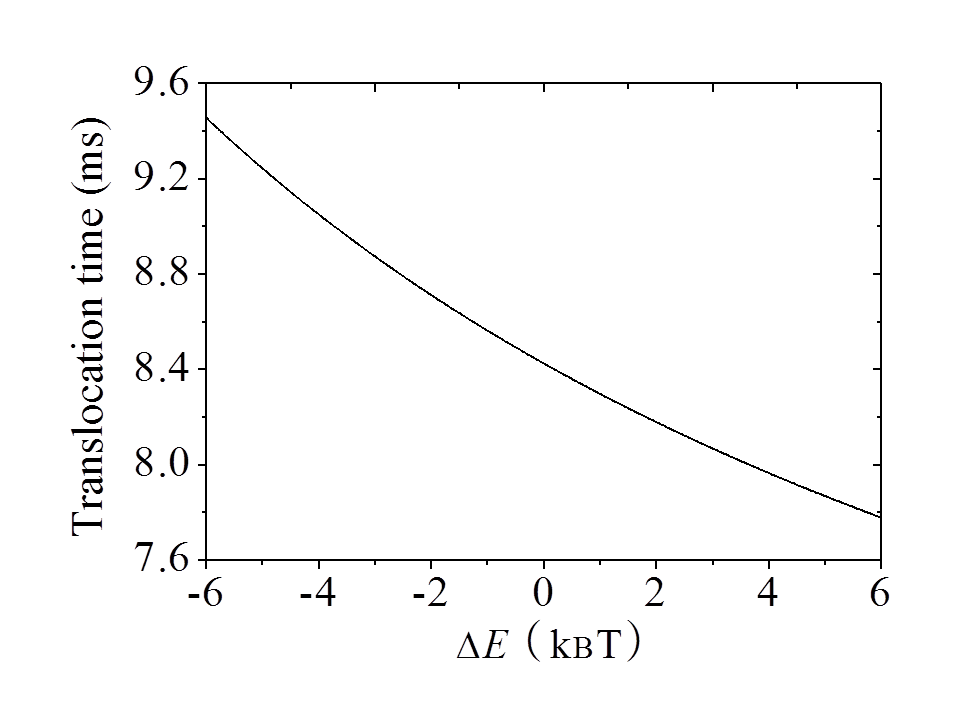

Supplement: Figure S4 — mRNA translocation time T 2 as a function of after the binding of EF-G.GTP for Case II that the ribosomal unlocking shifts the equilibrium toward the ratcheted conformation, as EF-G.GTP state does. (TIF) [file pone.0070789.s004.tif]

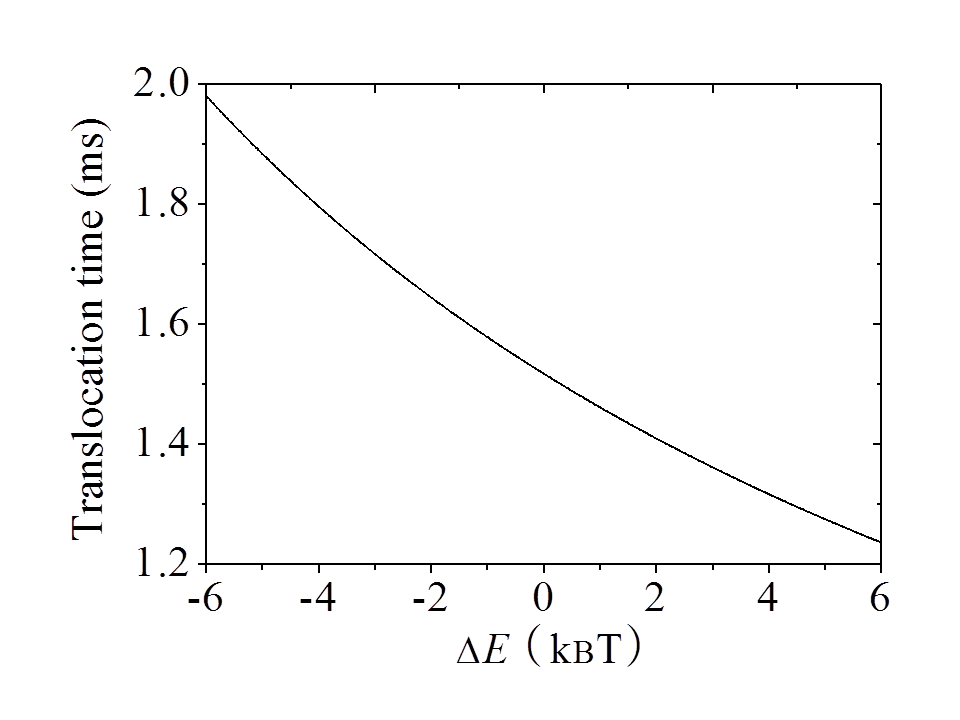

Supplement: Figure S5 — mRNA translocation time T 2 as a function of after the binding of EF-G.GTP for Case III that the ribosomal unlocking shifts the equilibrium toward the non-ratcheted conformation, which is contrary to Case II. (TIF) [file pone.0070789.s005.tif]

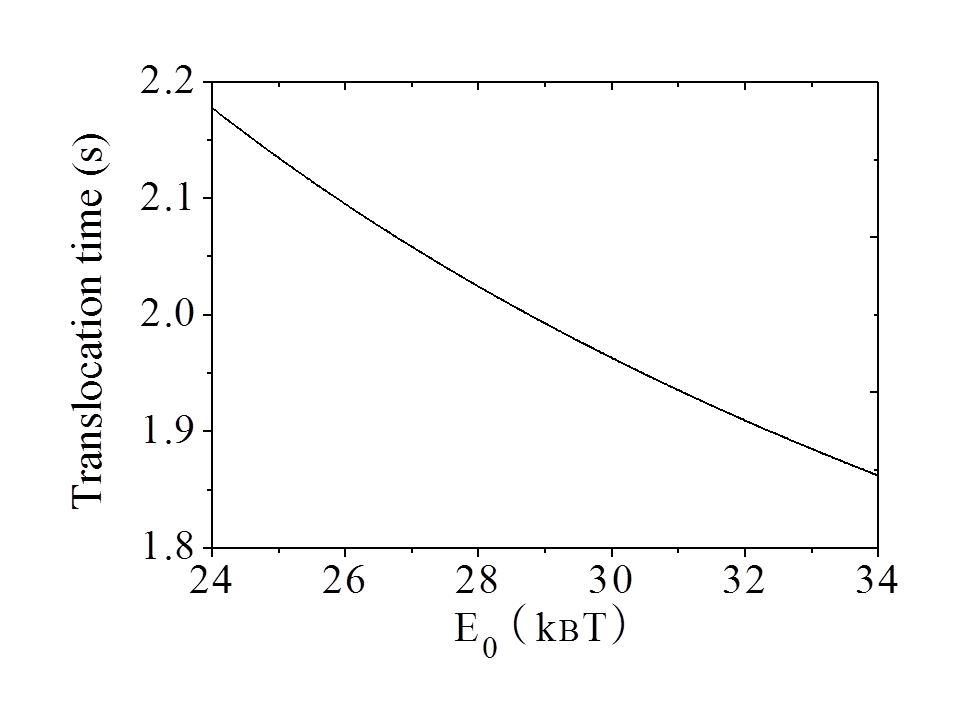

Supplement: Figure S6 — mRNA translocation time T 1 as a function of E 0. ENR = 23.02 kBT , EH = 26.54 kBT and EPOST = 23.33 kBT . (TIF) [file pone.0070789.s006.tif]

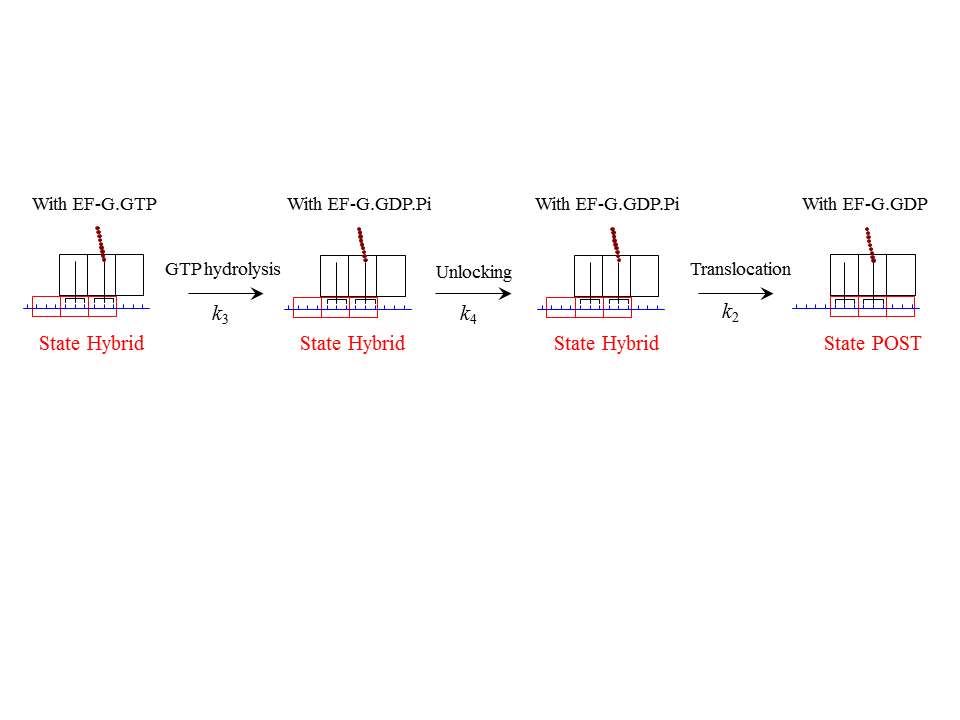

Supplement: Figure S7 — Kinetic scheme of EF-G.GTP-catalyzed mRNA translocation. (TIF) [file pone.0070789.s007.tif]

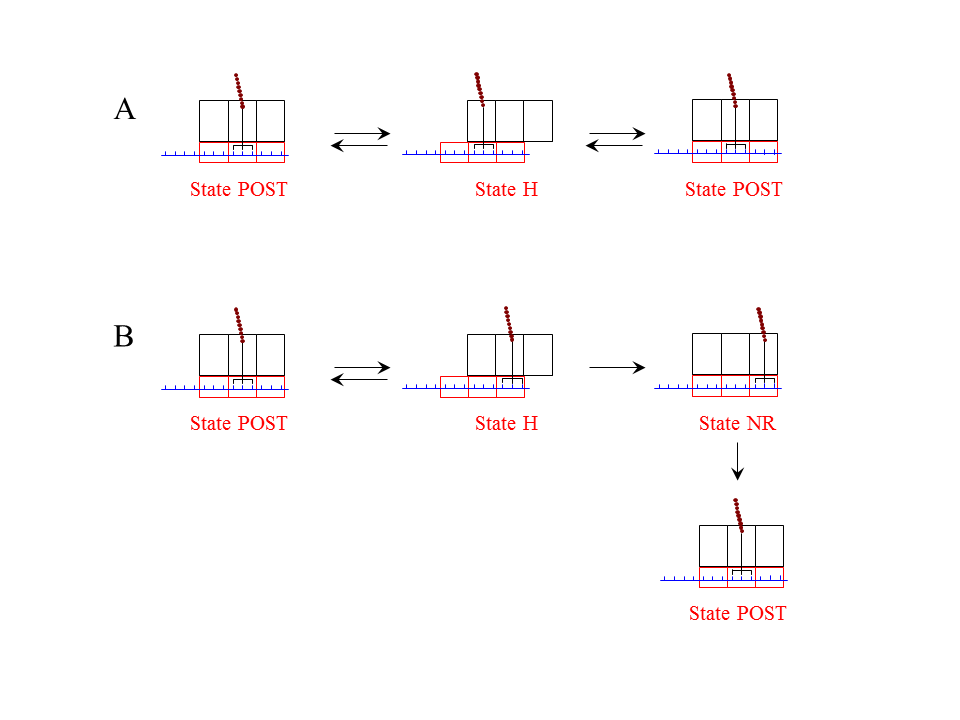

Supplement: Figure S8 — Schematic illustrations of two possible cases for backward translocation when only peptidyl-tRNA is bound to the P site. (TIF) [file pone.0070789.s008.tif]
