# Supplementary material for: Dynamics of Forward and Backward Translocation of mRNA in the Ribosome
Source: PLoS One. 2013 Aug 9;8(8):e70789. doi: 10.1371/journal.pone.0070789 (PMC3739767; doi:10.1371/journal.pone.0070789)
Supplement: Text S1 — Effect of variations of ENR and EH on forward mRNA translocation time. (DOC) [file pone.0070789.s009.doc]

**Text S1. Effect of variations of *E_NR_* and *E_H_* on forward mRNA translocation time**

To see the effect of variations of *E_NR_* and *E_H_* on mRNA translocation time, we take $E'_{NR}=E_{NR}+\Delta E$ and $E'_{H}=E_{H}+\Delta E$, where *E_NR_* and *E_H_* have the values as given in the main text and $\Delta E$ represents the variation. These variations of *E_NR_* and *E_H_* do not affect the probability of classical non-ratchet or hybrid state but changes significantly the rate of transition from non-ratchet to hybrid state and that of reverse transition.

**In the absence of EF-G**

With $E'_{NR}$ = 23.87*k_B_T* +$\Delta E$, $E'_{H}$ = 24.24*k_B_T* +$\Delta E$ and *E_POST_* = 33.91*k_B_T* (see Table 1), using Eq. (5) the calculated results of the spontaneous mRNA translocation time *T*_1_ as a function of $\Delta E$ are shown in Figure S1. It is seen that by decreasing $\Delta E$ from 0 to $-$6*k_B_T* the spontaneous translocation time *T*_1_ increases from about 1$\times10^{5}$ s to 1.24$\times10^{5}$ s.

**The binding of EF-G.GDPNP**

With $E'_{NR}$ = 23.02*k_B_T* +$\Delta E$, $E'_{H}$ = 26.54*k_B_T* +$\Delta E$ and *E_POST_* = 23.33*k_B_T* (see Table 1), using Eq. (7) the calculated results of the mRNA translocation time *T*_2_ as a function of $\Delta E$ are shown in Figure S2. It is seen that by decreasing $\Delta E$ from 0 to $-$6*k_B_T* the translocation time *T*_2_ increases from about 2 s to 2.28 s.

**The binding of EF-G.GTP**

*Case I*. With $E'_{NR}$ = 23.87*k_B_T* +$\Delta E$, $E'_{H}$ = 24.24*k_B_T* +$\Delta E$ and *E_POST_* = 15.24*k_B_T* (see Table 1), using Eq. (7) the calculated results of the mRNA translocation time *T*_2_ as a function of $\Delta E$ are shown in Figure S3. It is seen that by decreasing $\Delta E$ from 0 to $-$6*k_B_T* the translocation time *T*_2_ increases from about 1.57 ms to 1.88 ms.

*Case II*. With $E'_{NR}$ = 23.02*k_B_T* +$\Delta E$, $E'_{H}$ = 26.54*k_B_T* +$\Delta E$ and *E_POST_* = 17.54*k_B_T* (see Table 1), using Eq. (7) the calculated results of the mRNA translocation time *T*_2_ as a function of $\Delta E$ are shown in Figure S4. It is seen that by decreasing $\Delta E$ from 0 to $-$6*k_B_T* the translocation time *T*_2_ increases from about 8.43 ms to 9.46 ms.

*Case III*. With $E'_{NR}$ = 24.72*k_B_T* +$\Delta E$, $E'_{H}$ = 21.94*k_B_T* +$\Delta E$ and *E_POST_* = 12.94*k_B_T* (see Table 1), using Eq. (7) the calculated results of the mRNA translocation time *T*_2_ as a function of $\Delta E$ are shown in Figure S5. It is seen that by decreasing $\Delta E$ from 0 to $-$6*k_B_T* the translocation time *T*_2_ increases from about 1.52 ms to 1.98 ms.
