# Supplementary material for: Dynamics of Forward and Backward Translocation of mRNA in the Ribosome
Source: PLoS One. 2013 Aug 9;8(8):e70789. doi: 10.1371/journal.pone.0070789 (PMC3739767; doi:10.1371/journal.pone.0070789)
Supplement: Text S2 — Effect of variation of E 0 on forward mRNA translocation time. (DOC) [file pone.0070789.s010.doc]

**Text S2. Effect of variation of *E*_0_ on forward mRNA translocation time**

To see the effect of *E*_0_ on mRNA translocation time, we take *E_NR_* = 23.02*k_B_T*, *E_H_* = 26.54*k_B_T* and *E_POST_* = 23.33*k_B_T*, corresponding to the binding of EF-G.GDPNP (see Table 1). Using Eq. (5) the calculated results of the mRNA translocation time *T*_1_ as a function of *E*_0_ are shown in Figure S6. It is seen that the variation of *E*_0_ has a small effect on the mean forward mRNA translocation time.
