# Supplementary material for: Dynamics of Forward and Backward Translocation of mRNA in the Ribosome
Source: PLoS One. 2013 Aug 9;8(8):e70789. doi: 10.1371/journal.pone.0070789 (PMC3739767; doi:10.1371/journal.pone.0070789)
Supplement: Text S3 — Backward translocation cannot occur or cannot be detected when only peptidyl-tRNA is bound to the P site. (DOC) [file pone.0070789.s011.doc]

**Text S3. Backward translocation cannot occur or cannot be detected when only peptidyl-tRNA is bound to the P site**

Consider that only peptidyl-tRNA is bound to the 30S P site, as shown in Figure S8a and Figure S8b (left). When the ribosome is transited from the non-ratchet to ratchet conformation in either the absence or the presence of LepA, two possible cases can occur. One case (Figure S8a) is that the mRNA-tRNA complex is fixed to the 30S subunit by the interaction between them, while the peptidyl-tRNA is changed from the P/P state (left, Figure S8a) to P/E state (middle, Figure S8a) via breaking the specific interaction of the 50S P site with the peptidyl-tRNA, and then the P/E state (middle, Figure S8a) returns to the P/P state (right or left, Figure S8a). As it is noted, no backward translocation occurs for this case.

Another case (Figure S8b) is that the peptidyl-tRNA is fixed to the 50S P site by the specific interaction between them, while the mRNA-tRNA complex is translocated by one codon (State H, Figure S8b) via breaking the interaction between the mRNA-tRNA complex and the 30S subunit. After transition to the hybrid state (State H, Figure S8b), since the peptidyl-tRNA is now bound to the 30S A site the ribosome becomes labile. Then the ribosome is transited easily from the hybrid state (State H, Figure S8b) to non-ratchet state (State NR, Figure S8b). As determined in the main text, in the labile state of non-ratchet ribosome the affinity of 30S subunit for the mRNA-tRNA complex composed of two tRNAs is larger than 23.87*k_B_T*. In State NR (Figure S8b), the affinity of 30S subunit for the mRNA-tRNA complex composed of only one tRNA should be smaller than that composed of two tRNAs. Using the similar equation to Eq. (3) we calculate that that even for the affinity of the ribosome for the mRNA-tRNA complex in State NR (Figure S8b) to be as large as 20*k_B_T* (noting that the 50S A site has weak affinity for the peptidyl-tRNA), the mean time for the mRNA-tRNA complex composed of only peptidyl-tRNA tRNA to translocate from the A/A state to P/P state is about 0.09 s. This implies that the mRNA-tRNA complex would quickly translocate from the A/A state (State NR, Figure S8b) to P/P state (State POST, Figure S8b), which is consistent with the experimental data showing that the A site of the ribosome cannot be occupied in a stable fashion without a tRNA in the adjacent P site (Rheinberger et al., 1981). Thus, the backward translocation is almost undetectable for this case.

Taken together, we show that in either the absence or the presence of LepA, the backward translocation cannot occur or is almost undetectable when only peptidyl-tRNA is bound to the P site, which is consistent with the experimental data (Shoji et al., 2006; Konevega et al., 2007; Qin et al., 2006).

**References**

Konevega A.L., Fischer N., Semenkov Y.P., Stark H., Wintermeyer W., Rodnina M.V. (2007). Spontaneous reverse movement of mRNA-bound tRNA through the ribosome. Nat. Struct. Mol. Biol. 14, 318–324.

Qin Y, Polacek N., Vesper O., Staub E., Einfeldt E., Wilson D.N., Nierhaus K.H. (2006) The highly conserved LepA is a ribosomal elongation factor that back-translocates the ribosome. Cell 127, 721–733.

Rheinberger H.-J., Sternbach H., Nierhaus K.H. (1981). Three tRNA binding sites on Escherichia coli ribosomes. Proc. Natl. Acad. Sci. USA 78, 5310–5314.

Shoji S., Walker S.E., Fredrick K. (2006). Reverse translocation of tRNA in the ribosome. Mol. Cell 24, 931–942.
